# Rare Histological Types of Breast Cancer: A Single-Center Experience

**DOI:** 10.1155/tbj/1179914

**Published:** 2025-03-09

**Authors:** Mehmet Furkan Sağdıç, Cihangir Özaslan

**Affiliations:** ^1^Department of Surgical Oncology, Ankara Etlik City Hospital, Ankara, Turkey; ^2^Department of Surgical Oncology, University of Health Sciences Ankara Oncology Training and Research Hospital, Ankara, Turkey

**Keywords:** breast cancer, rare breast cancer, histological types

## Abstract

**Background:** Breast carcinoma is divided into at least 21 separate histologies, according to the 2019 World Health Organization (WHO) classification. The present study is dedicated to a 5% or rarer group of all breast cancer cases.

**Method:** In this study, we retrospectively considered the data of 4550 patients operated on for breast carcinoma at the Ankara Oncology Training and Research Hospital of the University of Health Sciences between January 2018 and February 2024. Of those cases, 401 were discovered to have rare breast cancer types. We also explored the cases by clinicopathological features, overall survival (OS), and disease-free survival (DFS).

**Results:** Our findings revealed a total of 10 rare breast cancer types in patients explored: mucinous carcinoma, micropapillary carcinoma, papillary group carcinomas, metaplastic carcinoma, neuroendocrine carcinoma, tubular carcinoma, cribriform carcinoma, apocrine carcinoma, acinic cell carcinoma, and secretory carcinoma. While mucinous, tubular, cribriform, papillary group carcinomas, micropapillary, and secretory carcinomas are described as types associated with good prognosis, metaplastic, neuroendocrine, apocrine, and carcinomas are described as types associated with relatively poor prognosis.

**Conclusion:** Scrutinizing the clinicopathological features of rare breast cancer types altogether may be the distinct contribution of this paper to the relevant literature and future research.

## 1. Introduction

Among the most prevalent women-specific malignancies, breast carcinoma demonstrates substantial variations by biological and morphological features. In this sense, breast carcinoma is divided into at least twenty-one separate histologies, according to the 2019 World Health Organization (WHO) classification [1]. The prevalent type is invasive ductal carcinoma nonspecific (IDC-NOS) type, accounting for 75% of all cases, and classical type invasive lobular carcinoma accounts for 10%–15% of breast cancer patients. However, the literature offers limited insights into or contradictory findings on clinical behavior and prognosis of rare histological subtypes due to their rare incidences [2, 3]. Thus, the present study is dedicated to a 5% or rarer group of all breast cancer cases.

Rare breast cancer types include carcinomas with good prognosis (e.g., mucinous carcinoma [2%–3%], tubular carcinoma [1%–2%], cribriform carcinoma [0.4%], papillary group carcinomas [0.5%], apocrine carcinoma [0.3%–0.4%], low-grade adenosquamous carcinoma [< 0.1%], and secretory carcinoma [< 1%]), with poor prognosis (metaplastic carcinoma [< 1%], neuroendocrine carcinoma [< 1%], micropapillary carcinoma [0.9%–2%], and with uncertain prognosis (e.g., mucinous cystadenocarcinoma [< 0.1%], fat-rich carcinoma [< 0.1%], and sebaceous carcinoma [< 0.1%]) [4]. It seems challenging for researchers to gather influential data on these rare histological subtypes, mandating extensive case series. In this regard, our research scrutinizes the clinicopathological features of patients with rare histological breast cancer to shed light on relevant clinical therapies and prognostic indicators.

## 2. Materials and Methods

In this study, we retrospectively considered the data of 4550 patients operated on for breast carcinoma at the Ankara Oncology Training and Research Hospital of the University of Health Sciences between January 2018 and February 2024. The data were extracted from the hospital database; incomplete pathological data and data of patients metastatic at the time of diagnosis were excluded from the study. Of those cases, 401 (8.8%) presented with the following rare breast cancer types: mucinous, micropapillary, papillary group carcinomas, metaplastic, neuroendocrine, tubular, apocrine, cribriform, acinic cell, and secretory carcinomas.

We accepted estrogen receptor (ER) and progesterone receptor (PR) positivity of ≥ 1% according to the 2010 American Society of Clinical Oncology/College of American Pathologists (ASCO/CAP) guideline [5]. Tumors were considered human epidermal growth factor receptor 2 (HER2) positive (HER2+) when HER2 average copies/cell was ≥ 6 and 3+ on immunohistochemical analysis, and gene amplification was present in the HER2/centromeric region of chromosome 17 (CEP17) ≥ 2 ratio by in situ hybridization [6]. Besides, Ki-67 staining was known to be performed using MIB-1 antibody (1:1000 titer) and Dako EnVision + Labeled Polymer.

Molecular subtypes were determined using immunohistochemistry (IHC), a rapid and widely utilized method. Luminal A is characterized by being ER and PR positive, HER2 negative, and having a low Ki-67 proliferation index. Luminal B is ER positive, usually PR negative or with low positive levels; HER2 status can be either positive or negative, and it has a high Ki-67 proliferation index. Triple negative (TN) is defined as ER, PR, and HER2 negative. HER2 positive refers to tumors that are HER2 positive but ER and PR negative. It is important to note that although IHC is a fast and common method, genetic and molecular analyses provide a more sensitive and comprehensive determination of the full biological profile of molecular subtypes [7].

For pathological staging, we considered the criteria proposed in the American Joint Committee on Cancer TNM Staging Guidelines (8th edition) [8]. We also noted variables age, radiological and pathological tumor sizes, tumor side, quadrant, grade, TNM classification, pathology (pure or mixed), ER and PR positivity, Ki-67 staining, HER2 positivity, molecular subtype, presence of lobular carcinoma in situ (LCIS) and ductal carcinoma in situ (DCIS), and type of surgery. Classification was made based on the dominant type in mixed pathology (> 50%). Breast-conserving surgery refers to conventional breast-conserving surgery along with techniques such as oncoplastic surgery, donut mastopexy (with upper and lower peduncles), parallelogram mastopexy, upper-external quadrantectomy (racquet), and batwing mastopexy.

We performed all statistical analyses on SPSS 25.0 (SPSS Inc., Chicago, Illinois, USA), and descriptives are presented as percentages, means, and medians. While we define the interval from diagnosis to mortality or last follow-up of any case as overall survival (OS), disease-free survival (DFS) indicates the interval between the surgery and follow-up of local recurrence and/or distant metastasis. If local recurrence and/or distant metastasis were not present, DFS refers to the interval between the surgery and the last follow-up. We generated survival curves using the Kaplan–Meier method.

## 3. Results

We present Table 1 for patients' clinical and pathological features, Table 2 for patients' surgery-specific conditions, and Table 3 for their OS and DFS. Clinicopathological and surgical specific conditions of papillary group carcinomas and metaplastic carcinoma subgroups are summarized in Tables 4 and 5.

### 3.1. Patients' Clinical and Pathological features (Table 1)

Our findings revealed a total of 10 rare breast cancer types in patients explored: mucinous (2.7%; *n* = 122), micropapillary (1.8%, *n* = 80), papillary group carcinomas (1.7%, *n* = 76), metaplastic (0.9%, *n* = 42), neuroendocrine (0.8%, *n* = 34), tubular (0.5%, *n* = 23), cribriform (0.3%, *n* = 14), apocrine (0.2%, *n* = 8), acinic cell (0.02%, *n* = 1), and secretory carcinomas (0.02%, *n* = 1).

While the oldest group consisted of papillary group carcinomas cases with a mean age of 65 years, the youngest was subsumed by tubular carcinoma cases with a mean age of 54 years. Besides, while the highest mean of radiological tumor size was calculated to be 37.45 mm in the metaplastic cancer group, the smallest mean (12.54 mm) was found in the tubular carcinoma group. Almost all cases in the tubular carcinoma group 91% (*n* = 21) were diagnosed at the T1 stage; however, all groups tended to be diagnosed at the T1 and T2 stages. While all patients in the apocrine carcinoma groups were observed with Grade 3 tumors, 95.2% (*n* = 40) in the metaplastic cancer group was Grade 3 carcinogenic, and all of the tubular cancer group was Grade 1 carcinogenic. The dominant molecular subtype was Luminal A in the mucinous (82%, *n* = 100), micropapillary (73.8%, *n* = 59), papillary group carcinomas (96%, *n* = 73), neuroendocrine (88%, *n* = 30), tubular (100%, *n* = 23), and cribriform carcinoma groups (85%, *n* = 12), but it was TN breast cancer in the metaplastic (85%, *n* = 36) and apocrine (50%, *n* = 4) carcinoma groups. Perineural invasion (23.8%, *n* = 19) and lymphovascular invasion (40%, *n* = 32) were the most prevalent in the micropapillary cancer group. While multifocality was the most prevalent in the tubular cancer group with 13% (*n* = 3), we discovered that the metaplastic cancer group was referred to neoadjuvant therapy (8 of 12 patients were identified as RCB-3, that is, unresponsive to treatment).

### 3.2. Patients' Surgery-Specific Conditions (Table 2)

Breast-conserving surgery rate was the highest in the tubular carcinoma group (65.2%, *n* = 15) and the lowest in the metaplastic cancer group (26.2%, *n* = 11). The mastectomy rate (with modified radical mastectomy) was found to be 73.8% in the metaplastic cancer group. The most and the least prevalent axillary dissection were in the micropapillary carcinoma group (62.5%, *n* = 50) and the papillary group carcinomas (13%, *n* = 10), respectively. Surgical margin positivity was the most prevalent in those who had undergone breast-conserving surgery (9.2%, *n* = 7) in the papillary group carcinomas. DCIS was found in six of these 7 patients. Sentinel lymph node positivity (41.3%, *n* = 33), axillary dissection rate (62.5%, *n* = 50), and extracapsular invasion (32.5%, *n* = 26) were the most common in the micropapillary cancer group.

### 3.3. Patients' Survival Status (Table 3)

The 5-year survival was uncovered to be 87.1% in the mucinous cancer group (*n* = 122). Distant metastasis was present in three patients (brain, vertebra, and supraclavicular lymph node metastases). While the patient with vertebral metastasis died in the 34th month of the treatment, the others were alive. Despite local recurrence in one patient, who had undergone a modified radical mastectomy, in the 7th month, the patient was recruited for re-excision and was alive at the 52nd month of their follow-up. In this group, we calculated OS to be 65.77 (62.01–69.53) months and DFS to be 63.88 (59.64–68.11) months. The 5-year survival was calculated to be 93% for 80 patients in the micropapillary cancer group. Four patients had distant metastasis (vertebra [1], liver [1], and lung [2] metastases). While one patient died in the 49th month due to liver failure emerging in the 33rd month, the others were being followed up. No local recurrence was present in patients, and we determined OS and DFS to be 62.49 (60.461–64.532) and 60.11 (56.862–63.365) months.

The papillary cancer group consisted of 76 patients with a 5-year survival of 85.4%. While OS was calculated to be 58.26 (54.647–61.878) months, DFS was 58.27 (54.676–61.881) months in the group. In the metaplastic cancer group (*n* = 42), 5-year survival was 70%, and OS and DFS were found to be 52.22 (44.428–60.016) and 50.42 (42.309–58.546) months. Local recurrence developed in three patients in the 2nd, 11th, and 39th months, and two were lost. Moreover, two other patients died due to lung metastasis in the 40th and 25th months, respectively.

A total of 34 patients were followed in the neuroendocrine cancer group with a 5-year survival of 82.6%. One patient developing lung metastasis in the 3rd month was lost. While OS was found to be 55.86 (49.339–62.381) months, DFS was 55.85 (49.321–62.385) months in the group. When it comes to the tubular cancer group (*n* = 23), all patients were alive in the 52nd month. One patient developed liver metastasis in the 16th month; nevertheless, no local recurrence was observed in the group. OS and DFS could not be calculated since all patients were alive.

One of eight patients in the apocrine cancer group died in the 10th month, and 80% were alive in the 45th month. No recurrence or metastasis was discovered in patients. OS and DFS were found to be 39.16 (28.730–49.604) months. Among 14 patients in the cribriform cancer group, one patient developed liver metastasis in the 23rd month. One patient died due to local recurrence in the 16th month. OS and DFS were found to be 58.30 (49.561–67.039) and 55.60 (46.603–64.597) months in the group.

The only patient in the acinic cell cancer group was followed up for 52 months without recurrence or metastasis. Finally, one patient in the secretory cancer group was followed up for 12 months without recurrence or metastasis at the time of this study.

### 3.4. Features of Papillary Group Carcinomas and Metaplastic Carcinoma Subgroups (Tables 4 and 5)

Papillary group carcinomas were classified according to the 5th classification of WHO 2019 [9]. 78.6% (*n* = 60) of 76 patients were detected as invasive papillary carcinoma, 19.7% (*n* = 15) as encapsulated papillary carcinoma with invasion, and 1.3% (*n* = 1) as intraductal papillary adenocarcinoma. In the invasive solid papillary cancer group, Grade 3 features were observed in 16% of patients, while this rate was observed as 6.7% in encapsulated papillary carcinoma. In the invasive solid papillary carcinoma subtype, the rate of Ki-67 > 15% was higher than in the encapsulated group (60%–46%). Lymphovascular invasion and perineural invasion were not observed in the encapsulated group, while they were observed in the invasive papillary group at 8.3% and 5%, respectively.

Metaplastic cancer subtypes are as follows: metaplastic carcinoma with mesenchymal differentiation (50%, *n* = 21), squamous cell carcinoma (33%, *n* = 14), spindle cell carcinoma (7%, *n* = 3), fibromatoid metaplastic carcinoma (4%, *n* = 2), and low-grade adenosquamous carcinoma (2%, *n* = 1) was classified as mixed metaplastic carcinoma (%2, *n* = 1) [10]. Squamous cell carcinoma had the largest tumor diameter compared to other subtypes (mean; 40.28 ± 18.20). While the T3-4 rate was 28% (*n* = 6) in metaplastic carcinoma with mesenchymal differentiation, it was 43% (*n* = 6) in squamous cell carcinoma. Lymphovascular and perineural invasion is higher in squamous cell carcinoma (4.8%–14.3%; 19%–28.5%). Axillary dissection rate in squamous cell carcinoma was 64% (*n* = 9), and metaplastic carcinoma with mesenchymal differentiation was 33% (*n* = 7). OS and DFS could not be calculated due to insufficient number of patients, but 5 patients with squamous cell carcinoma, 2 patients with carcinoma with mesenchymal differentiation, 1 patient with spindle cell carcinoma, and 1 patient with mixed metaplastic carcinoma died.

## 4. Discussion

Research on rare histopathological types of breast cancer often relies on case reports and series with an insufficient number of patients. In this sense, our research can be considered among rare studies scrutinizing the clinicopathological features of and survival in each rare type of breast cancer under the same umbrella. Accordingly, a comprehensive summary of clinicopathological features pertinent to 13 rare breast cancer types will likely contribute to the relevant literature and future research. In addition, we are able to present the clinical features of cases with acinic cell, and secretory carcinomas—truly scarce types reported in the literature.

Mucinous carcinoma accounts for 2%-3% of breast cancers [11]. Similarly, we discovered its incidence to be 2.7% (*n* = 122). This type tends to occur at older ages, and the mean age of diagnosis was reported to be 71 years [12]. In their study, Lu et al. examined 4578 cases and reported the mean age for mucinous carcinoma to be 65 years [13]. In our study, the median age for this type was calculated to be 63.5 years. This type is also characterized by frequent ER and PR expression and HER2 negativity [12], implying that the molecular subtype is more likely to be Luminal A. Accordingly, Luminal A was found to be 82% (*n* = 100) among cases with mucinous carcinoma. Besides, while Lu et al. reported that 56% of patients presented with Grade 1% and 34.3% had Grade 2 tumors, we found these rates to be 12.3% (*n* = 15) and 64.8% (*n* = 79), respectively, in this group. In parallel, the mentioned study revealed T1 and T2 as 66.6% and 27.6%, respectively, and these rates became 35.2% (*n* = 43) and 47.5% (*n* = 58) in this study, respectively. Mucinous cancer typically presents at an older age, with a higher T stage and larger tumor size compared to NOS. However, it shows lower regional lymph node involvement, lower histological grade, and lower TNM stage, indicating less aggressive behavior [14]. We found the 5-year survival of this group to be 87.1%, overlapping with the findings of Marrazzo et al., who reported 5-year survival to be 92% among their patients [15]. Finally, while Di Saverio et al. calculated distant metastasis to be 2% among 11,422 patients [12], we discovered distant metastases in 2.45%.

Accounting for 0.9%–2.0% of all breast cancer cases [15], micropapillary carcinoma is a distinct, aggressive histological variant of breast cancer with a higher prevalence of local recurrence, lymph node metastasis, lymphovascular invasion, and distant metastasis [16, 17]. The mean age of diagnosis of this type was previously reported to be between 50-60 years [18–21]. Our findings demonstrated that 1.76% (*n* = 80) of cases had invasive micropapillary carcinoma and that the mean age at diagnosis was 57 years. The literature adequately informs that invasive micropapillary carcinomas present with larger tumors at diagnosis [18], which may be attributed to the rapid growth pattern of this type of cancer [19]. In their study, Zekioglu et al. also reported the tumor size in invasive micropapillary carcinoma as 34 mm [22]. In this study, we calculated the mean tumor size to be 35.83 mm, the largest tumor size following the mean tumor size in the metaplastic carcinoma group. Besides, Lewis et al. reported 75.3% of the micropapillary carcinoma cases as Luminal A, 14.8% as Luminal B, 4.7% as HER2 positive, and 5.2% as TN [20]. Overlapping with this finding, we found these rates to be 73.8% (*n* = 59), 22.5% (*n* = 18), 2.5% (*n* = 2), and 1.2% (*n* = 1), respectively, among our micropapillary carcinoma cases.

The significant aspect of invasive micropapillary carcinoma is increased lymphovascular invasion and lymph node involvement [21]. A previous study documented axillary lymph node involvement in 70% of patients [22]. In their research, Lewis et al. retrospectively examined the data of 2660 invasive micropapillary carcinoma patients and concluded regional lymph node metastasis to be 55.2% [20]. Similar to this finding, we found it to be 62.5% (*n* = 50). Lymphovascular invasion, on the other hand, was previously reported to be 69% by Jones et al. and 70.9% by Nangong et al. in micropapillary carcinoma patients [16, 17]. In our study, lymphovascular invasion was located in 40% (*n* = 32) of the patients. Although this rate is higher when compared to the other types, it remains lower than the findings of the mentioned research, which may be due to a general tendency to omit lymphovascular invasion indicators in pathology reports. While sentinel lymph node positivity was found to be 60% by Solà et al. and 70% by Lyu et al. [23, 24], it was 41.3% (*n* = 33) in our micropapillary carcinoma group—the highest compared to the other groups. Moreover, it is known that micropapillary carcinomas are present with high-grade tumors. Accordingly, similar to the study by Li et al. (62.71%), we found that 61.2% (*n* = 49) of patients in this group were with Grade 3 tumors [25]. Micropapillary cancer is characterized by a higher rate of lymphovascular invasion and lymph node metastasis compared to IDC-NOS. They usually present as larger tumors with higher histologic grades and a higher proportion of disease-positive lymph nodes [26, 27]. Gao et al. reported the mean Ki-67 staining to be 35.8, and it was 39.55 in our study [28]. Median follow-up time and OS were reported to be 64 months and 91.2%, respectively, by Chen et al. and 51 months and 93.5%, respectively, by Chen et al. [29, 30]. In this study, we found 5-year survival to be 93%. We also discovered no recurrence in this group but distant metastasis in only four patients.

Papillary group carcinomas usually accounts for 1%–2% of breast cancers [31] and was calculated as 1.67% (*n* = 76) among our patients. It tends to be detected more in postmenopausal older adult women [32–34]. Consistent with the literature, the mean age of diagnosis of this type was 65 years in this study, making this group the oldest one among the others. As a rule of thumb, papillary group carcinomas are accompanied by high ER and PR positivity and low c-erbB-2 negativity. In one of the most extensive series in the literature with 524 invasive papillary carcinoma cases, Zheng, Hu, and Shao reported that this type exhibits an earlier T stage than its NOS type, low-grade and decreased lymph node involvement, higher ER and PR positivity, and lower HER2 amplification [35]. In our study, ER positivity was 100% (*n* = 76), PR positivity was 94.7% (*n* = 72), and HER2 negativity was 96.1% (*n* = 73). While lymph node metastasis was reported at a rate of 11.6% in the study above, we discovered it to be 11.8% and the rate of axilla dissection to be 13.2%. Chen et al. found that T1 (68.7%) and T2 (23%) patients constituted 92% of the 348 invasive papillary cancer cases explored [36]. In our study, these rates became 48.8% (*n* = 37) for T1 and 38.1% (*n* = 29) for T2. Interestingly, while the cases were dominantly staged as T1, the rate of T2-staged invasive papillary carcinoma cases was slightly higher in this study, which may highlight the significant need for enhancing screening programs in our country. While the rate of Luminal A subtype was noted as 88.2% in the study by Chen et al., it was 96.1% in our study. About half of the cases with encapsulated papillary carcinoma are associated with DCIS or invasive carcinoma, and the tumor side involves the potential for widespread DCIS [37]. In this regard, the most prevalent surgical margin positivity (DCIS positivity in six of 7 patients) and reoperation rate belonged to this group with 9.2%. In addition, in the subgroup analysis, we observed higher surgical margin positivity rates in the encapsulated papillary cancer group than in the invasive solid papillary group (20%–5%). In papillary group carcinomas surgery, surgeons may need to keep surgical margins wide, adopt an appropriate oncoplastic technique, or consider mastectomy and reconstruction.

In their study, Chen et al. reported the median follow-up time to be 41 months and the 5-year survival rate to be 99.7%. These indicators became 58 months and 90%, respectively, among patients without any recurrence or distant metastasis. The low OS in our study may be because some of the patients were lost due to COVID-19 pandemic-related complications following surgery.

Metaplastic carcinoma is a rare breast cancer type with a remarkably poor prognosis and accounts for 0.2%–5% of all breast cancers [38]. In this study, we found its prevalence to be 0.92%. While Corso et al. reported the median age of diagnosis to be 55 years (46–66 years), it was discovered to be 53 years (34–84 years) in our study [39]. Compared to the other rare types, we calculated the pathological tumor size in this type to be significantly higher with 36.97 mm, but it seems consistent with what was previously reported in the literature [39, 40]. The rate of T2–T3-stage tumors was revealed to be 63.2% (*n* = 153) and 69.1% (*n* = 217) in the studies by Corsa et al. [39, 40], and it was slightly higher in our study with 85.8% (*n* = 42), which may be due to our small sample size or patients presenting at a later stage. Concerning tumor grade, while Corsa et al. detected 95.3% of patients to present with Grade 3 tumors, it was 95.2% in our study. Similarly, 85.7% (*n* = 36) of our patients were TN, overlapping with the literature [39, 40]. Besides, the highest mastectomy rate was discovered in this group, 73.8% (*n* = 31), which may be associated with large tumor sizes in presentation and surgeons' tendency to adopt more aggressive treatment modalities for metaplastic carcinoma patients. It should be noted that while mastectomy rates vary between 25%-92.7% in the literature, most previous studies reported that it is significantly higher [41]. It is usually characterized by advanced age, larger tumor sizes (> 2 cm), higher grade (Grade 3), and TN phenotype but can also be hormone receptor (HR) positive or HER2 positive. All molecular subtypes of metaplastic carcinoma, including HR positive, HER2-positive, and TN, have a worse prognosis compared to NOS and recurrence is more frequent [42].

Metaplastic breast cancer is divided into 7 subtypes according to the WHO classification [10]. While the form with the best prognosis is the type showing mesenchymal differentiation [43], the most aggressive form is squamous cell carcinoma [44]. In the study of Tadros et al., which is one of the largest metaplastic cancer series in the literature, higher pathologic tumor stage (T3/4) and nodal stage (N2/3) as well as the presence of lymphovascular invasion were associated with poorer OS and DFS, and these were observed to be high in the squamous form [44]. In our study, the tumor diameter was the largest, lymphovascular and perineural invasion, and axillary dissection rate was the most frequent in squamous cell carcinoma compared to other subtypes.

The group that was the most frequently referred to neoadjuvant therapy included patients with metaplastic carcinoma with 28.6% (*n* = 12). Residual cancer burden system (RCB) count, Wong et al. identified 1 (5%) patient as RCB-1, 17 (81%) as RCB-2, and 3 (14%) as RCB-3 [45]. In other words, metaplastic carcinoma has an insufficient response to neoadjuvant therapy. Similarly, this group was the least responsive to neoadjuvant treatment among the others; that is, most of the patients in this group (*n* = 8, 66.6%) were referred to neoadjuvant therapy and identified as RBC-3. In their study, Yu et al. calculated the median follow-up time to be 51 months and local recurrence to be 7.6 months [46]. Corso et al. reported 5-year survival rate as 93.7% [39]. Likewise, we computed the median OS as 52 months and 5-year survival as 70% for patients in this group. In this regard, the metaplastic carcinoma group had the highest rate of local recurrence with 7.1% (*n* = 3).

Neuroendocrine carcinoma is often detected in 0.1%–5% of breast cancer cases [47], and its prevalence was found to be 0.74% (*n* = 34) in this study. While the mean age of diagnosis is reported between 60 and 70 in the literature [48], we discovered it to be 63 years. Moreover, its prognosis is accompanied by ER and PR positivity and HER2 negativity [48, 49]. In this study, the dominant molecular subtype of neuroendocrine carcinoma became Luminal A (88.2%), along with Luminal B (11.8%). Comparing 142 neuroendocrine carcinomas with IDCs, Wang et al. noted significantly lower OS (26 vs. 34 months), a higher tumor grade (Grade 3 = 42%), a larger mean tumor size (32 mm), and regional node positivity (43%) in the neuroendocrine carcinoma group [48]. These findings then seem to be consistent with our results (Grade 3 = 64%, *n* = 22; mean tumor size = 31.29 mm; axilla node positivity = 38.2%, *n* = 13). Moreover, 5-year survival was 82% in the neuroendocrine carcinoma group, making it the second worst survival group following the metaplastic carcinoma group (55.86, 49.339–62.381) months.

Compared with tubular carcinoma NOS, it is characterized by smaller tumor size, less lymph node involvement, and a predominantly Luminal A molecular profile and is a subgroup with an excellent prognosis with low recurrence rates [50–52]. Although tubular carcinoma accounts for approximately 1%-2% of all breast cancers, it was calculated to be 0.5% (*n* = 23) among our patients. It is often observed in postmenopausal women and at a mean age of 57 ± 13.8 years [53]. In this study, the mean age of diagnosis was 54.43 ± 10.23 years. Consistent with the literature, we identified 91% (*n* = 21) of the patients in T1 stage [54]. Low-grade DCIS is known to be associated with tubular carcinoma [55], and similarly, we found this type to be associated with DCIS, the most following apocrine carcinoma (65%).

This type is characterized by Grade 1 tumors with strong ER and PR positivity and HER2 negativity [54] and often shows Luminal A pattern [56]. Likewise, we found that all patients in this group to exhibit Luminal A pattern and to be with Grade 1 tumors. Besides, 10%–56% of these tumors are multifocal in the same breast, and the most prevalent multifocality was detected in this group with 13% (*n* = 3). In their study, Lam et al. reported lymph node positivity to be 17.8% and the rate of breast-conserving surgery to be 35.6% in this group [57]. We caught the dissection rate due to axilla positivity to be 17.4% (*n* = 4), and the highest number of patients undergoing breast-conserving surgery (65%) in the tubular carcinoma group. Tubular carcinoma patients often experience low local recurrence and distant metastasis. For example, Javid et al. found local recurrence to be < 1% and no distant metastasis among 111 tubular cancer cases during the 72-month follow-up [58]. Although OS could not be calculated due to the insufficient number of patients in this group, the 5-year survival was 100%. No local recurrence was detected; however, liver metastasis developed in one patient in the 16th month.

Cribriform carcinoma is attributed to 0.4% of all breast cancers, is associated with a good prognosis, and is usually present with ER and PR positivity and HER2 negativity [59, 60]. The cribriform carcinoma subsumed 0.3% (*n* = 14) of patients, and all cribriform carcinoma patients in this study were ER positive, except for one PR-negative and one HER2-positive case. Besides, while Ki-67 was previously reported to be ≤ 14 in 72% of the cases [59], we found it to be 36% (*n* = 5), which may be explained by the small sample size in this study or the differences in antibody clones for Ki-67 staining. On the other hand, Xi-Yu et al. noted 85% of 618 cribriform carcinoma patients with Grade 1 and 2 tumors, and this rate was 92% (*n* = 13) in this study. In the same study, almost all patients were staged as T1 and T2 (97%), which exactly matches our finding (97%). Moreover, the authors compared cribriform and ductal carcinomas and found better survival in the cribriform carcinoma group (95.3%–90.1%) [61]. Cribriform carcinoma is generally seen as a smaller, lower stage, HR-positive tumor compared to NOS. Cribriform breast cancer is associated with a lower rate of metastasis and recurrence than NOS [52]. We found the median follow-up time in the cribriform carcinoma group to be 58 months, but one out of 14 patients was lost in the 16th month due to local recurrence.

The incidence of apocrine carcinoma varies between 0.3%-1% [62, 63], and it was uncovered to be 0.18% (*n* = 8) in our study. This type often has high-grade cytological features and is usually accompanied by DCIS [64]. In this study, DCIS association was found to be the second most prevalent in this group with 87.5% (*n* = 7) after cribriform carcinoma. Apocrine carcinoma typically exhibits the feature of TN [63]. While Saridakis et al. reported that half of 76 apocrine carcinoma patients were TN [65], this rate was 54% (*n* = 4) in our study. In their research, İmamovic et al. examined 62 apocrine carcinoma patients, and their findings are quite similar to our results [66] (e.g., mean age = 59.5 years vs. 59.1 years; T2 stage = 54.5% vs. 54.8%; Grade 3 tumor = 72% vs. 54.8%; mastectomy rate = 67.7% vs. 68.2%; 5-year OS = 70.2% vs. 68.2%). The prognosis of apocrine carcinoma is similar to NOS. In addition, TN apocrine carcinoma is known to have a better prognosis compared to NOS TN breast cancers [67].

As a final note, while mucinous, tubular, cribriform, and papillary group carcinomas, as well as apocrine and secretory carcinomas, are associated with a good prognosis, metaplastic, neuroendocrine, and micropapillary carcinomas are considered to have a relatively poor prognosis [4]. In contrast, we discovered a worse prognosis in the apocrine carcinoma group and a relatively better prognosis in the micropapillary carcinoma group, which may probably be due to the insufficient number of patients in these groups in this study.

The limitations of this study include its retrospective design, single-center setting, and the absence of a matched IDC-NOS comparative analysis. In the future, we hope to conduct a larger series and a multicentric study that includes an IDC-NOS comparative analysis.

## 5. Conclusion

Overall, the present research can be counted among rare studies extensively investigating the clinicopathological features of rare breast cancer types. In this sense, we believe that it brings a substantial contribution to the relevant literature and guides future research. Interpretation of these features can provide guidance regarding the extent, type, and reconstruction options of surgery to be applied. Yet, it should be noted that prospective research involving a larger sample size and long-term follow-ups are still needed.

## Figures and Tables

**Table 1:** Patients' clinical and pathological features.

Clinicopathological variables	Mucinous (*n* = 122)	Micropapillary (*n* = 80)	Papillary group carcinomas (*n* = 76)	Metaplastic (*n* = 42)	Neuroendocrine (*n* = 34)	Tubular (*n* = 23)	Cribriform (*n* = 14)	Apocrine (*n* = 8)	Acinic cell (*n* = 1)	Secretory (*n* = 1)
*Age*										
Mean	62.07 ± 12.16	57.23 ± 13.43	65.3 ± 13.46	56.4 ± 13.76	63.26 ± 14.99	54.43 ± 10.23	55.93 ± 10.43	61.25 ± 14.69	45	63
Median	63.5 (36–88)	56.5 (33–84)	66 (28–89)	53 (34–84)	61 (30–88)	52 (42–81)	50.5 (40–75)	60 (37–83)		

Radiological tumor size (mean-mm-sd)	25.21 ± 12.88	24.56 ± 14.81	25.48 ± 15.02	37.45 ± 17.34	27.17 ± 14.80	12.54 ± 5.07	23.66 ± 9.24	24.85 ± 15.89	25	29

Pathological tumor size (mean-mm-sd)	31.65 ± 21.32	35.83 ± 28.58	27.67 ± 21.11	36.97 ± 11.80	31.29 ± 18.10	10.52 ± 5.62	22.07 ± 9.26	31.25 ± 23.29	60	29

*T stage*										
Tis	—	—	—	—	—	—	—	1 (12.5%)	—	—
T1	43 (35.2%)	23 (28.7%)	37 (48.8%)	5 (11.9%)	13 (38.3%)	21 (91.3%)	6 (42.9%)	2 (25%)	—	—
T2	58 (47.5%)	41 (51.2%)	29 (38.1%)	23 (54.8%)	15 (44.1%)	2 (8.7%)	8 (57.1%)	3 (37.5%)	—	1 (100%)
T3	21 (17.2%)	15 (18.8%)	10 (13.1%)	13 (31%)	5 (14.7%)	—	—	2 (25%)	1 (100%)	—
T4	—	1 (1.1%)	—	1 (2.4%)	1 (2.9%)	—	—	—	—	—

*Pure or mix pathology*										
Pure	95 (77.9%)	38 (47.5%)	68 (89.5%)	39 (92.9%)	33 (97.1%)	19 (82.6%)	9 (64.3%)	8 (100%)	1 (100%)	1 (100%)
Mix	27 (22.1%)	42 (52.5%)	8 (10.5%)	3 (7.1%)	1 (2.9%)	4 (17.4%)	5 (35.7%)	—	—	—

*Grade*										
Grade 1	15 (12.3%)	4 (5%)	9 (11.8%)	—	1 (2.9%)	23 (100%)	9 (64.3%)	—	—	—
Grade 2	79 (64.8%)	27 (33.8%)	56 (73.7%)	2 (4.8%)	11 (32.4%)	—	4 (28.6%)	—	1 (100%)	1 (100%)
Grade 3	28 (23%)	49 (61.2%)	11 (14.5%)	40 (95.2%)	22 (64.7%)	—	1 (7.1%)	8 (100%)	—	—

*Hormone receptor*										
Negative	5 (4.1%)	2 (2.5%)	1 (1.3%)	1 (2.4%)	0	1 (4.3%)	—	6 (75%)	1 (100%)	1 (100%)
Positive	117 (95.9%)	78 (97.5%)	75 (98.7%)	41 (97.6%)	34 (100%)	22 (95.7%)	14 (100%)	2 (25%)	—	—

*Estrogen receptor*										
Negative	4 (3.3%)	4 (5%)	—	1 (2.4%)	0	0	—	6 (75%)	1 (100%)	1 (100%)
Positive	118 (96.7%)	76 (95%)	76 (100%)	41 (97.6%)	34 (100%)	23 (100%)	14 (100%)	2 (25%)	—	—

*Progesterone receptor*										
Negative	16 (13.1%)	6 (7.5%)	4 (5.3%)	1 (2.4%)	1 (2.9%)	0	1 (7.1%)	6 (75%)	1 (100%)	1 (100%)
Positive	106 (86.9%)	74 (92.5%)	72 (94.7%)	41 (97.6%)	33 (97.1%)	23 (100%)	13 (92.9%)	2 (25%)	—	—

Ki-67 (mean-sd)	23.76 ± 14.58	39.55 ± 19.76	24.21 ± 17.25	70.95 ± 19.48	35.85 ± 20.18	10.52 ± 7.69	25.79 ± 19.93	43.75 ± 23.10	50	30

Ki-67										
≤ 15	42 (34.43%)	6 (7.5%)	28 (36.8%)	0	4 (11.8%)	18 (78.3%)	5 (35.7%)	—	−1 (100%)	—
> 15	80 (65.57%)	74 (92.5%)	43 (56.6%)	42 (100%)	30 (88.2%)	5 (21.7%)	9 (64.3%)	8 (100%)	—	1 (100%)
Missing			5 (6.6%)				—			—

*HER2/neu status*										
Negative	103 (84.4%)	60 (75%)	73 (96.1%)	36 (85.7%)	30 (88.2%)	22 (95.7%)	13 (92.9%)	5 (62.5%)	1 (100%)	1 (100%)
Positive	19 (15.6%)	20 (25%)	3 (3.9%)	6 (14.3%)	4 (11.8%)	1 (4.3%)	1 (7.1%)	3 (37.5%)	—	—

*Molecular subtypes*										
Luminal A	100 (82%)	59 (73.8%)	73 (96.1%)	1 (2.4%)	30 (88.2%)	23 (100%)	12 (85.8%)	1 (12.5%)	—	—
Luminal B	18 (14.8%)	18 (22.5%)	3 (3.9%)	1 (2.4%)	4 (11.8%)	0	1 (7.1%)	1 (12.5%)	—	—
HER2 positive	1 (0.8%)	2 (2.5%)	0	4 (9.5%)	0	0	—	2 (25%)	—	—
Triple negative	3 (2.5%)	1 (1.2%)	0	36 (85.7%)	0	0	1 (7.1%)	4 (50%)	1 (100%)	1 (100%)

*LCIS*										
No	118 (96.7%)	74 (92.5%)	72 (94.7%)	39 (92.9%)	33 (91.1%)	20 (87%)	13 (92.9%)	8 (100%)	1 (100%)	1 (100%)
Yes	4 (3.3%)	6 (7.5%)	4 (5.3%)	3 (7.1%)	1 (2.9%)	3 (13%)	1 (7.1%)	—	—	—

*DCIS*										
No	60 (49.2%)	41 (51.2%)	51 (67.1%)	27 (64.3%)	18 (52.9%)	8 (34.8%)	3 (21.4%)	1 (12.5%)	—	1 (100%)
Yes	62 (50.8%)	39 (48.8%)	25 (32.9%)	15 (35.7%)	16 (47.1%)	15 (65.2%)	11 (78.6%)	7 (87.5%)	1 (100%)	—

*Perineural invasion*										
No	116 (95.1%)	61 (76.2%)	73 (96.1%)	39 (92.9%)	33 (97.1%)	21 (91.3%)	13 (92.9%)	8 (100%)	1 (100%)	1 (100%)
Yes	6 (4.9%)	19 (23.8%)	3 (3.9%)	3 (7.1%)	1 (2.9%)	2 (8.7%)	1 (7.1%)	—	—	—

*Lymphovascular invasion*										

No	106 (86.90%)	48 (60%)	73 (96.1%)	32 (73.2%)	27 (79.4%)	20 (87%)	12 (85.7%)	7 (87.5%)	—	—
Yes	16 (13.1%)	32 (40%)	3 (3.9%)	10 (23.8%)	7 (20.6%)	3 (13%)	2 (14.3%)	1 (12.5%)	1 (100%)	1 (100%)

*Synchronicity*										
No malignancy in the contralateral breast	118 (96.70%)	75 (93.75%)	74 (97.4%)	41 (97.6%)	31 (91.2%)	21 (91.3%)	14 (100%)	8 (100%)	1 (100%)	1 (100%)
Malignancy in the contralateral breast	4 (3.3%)	5 (6.25%)	2 (2.6%)	1 (2.4%)	3 (8.8%)	2 (8.7%)	—	—	—	—

*Metachronous*										
No malignancy in the contralateral breast	122 (100%)	80 (100%)	76 (100%)	42 (100%)	34 (100%)	22 (95.7%)	14 (100%)	8 (100%)	1 (100%)	1 (100%)

There is malignancy in the contralateral breast	—	—	—	—	—	1 (4.3%)	—	—	—	—

*Disease focality*										
Unifocal	108 (88.5%)	72 (90%)	67 (88.2%)	42 (100%)	32 (94.1%)	20 (87%)	14 (100%)	8 (100%)	1 (100%)	1 (100%)
Multifocal	14 (11.5%)	8 (10%)	9 (11.8%)	0	2 (5.9%)	3 (13%)	—	—	—	—

*Multifocal pathology*										
Same	6 (4.91%)	4 (5%)	—	—	—	—	—			
NST	5 (4.09%)	4 (5%)	7 (9.21%)		2 (5.88%)	3 (13.04%)	—	—	—	
Other	3 (2.45%)		2 (2.63%)		—	—		—		

*Neoadjuvant status*										
No	97 (79.5%)	64 (80%)	73 (96.1%)	30 (71.4%)	31 (91.2%)	23 (100%)	12 (85.7%)	7 (87.5%)	1 (100%)	1 (100%)
Yes	25 (20.5%)	16 (20%)	3 (3.9%)	12 (28.6%)	3 (8.8%)	—	2 (14.3%)	1 (12.5%)	—	—

*Residual cancer burden system*										
1	6 (4.91%)			1 (2.38%)	2 (5.88%)	—	—	1 (12.5%)		
2	8 (6.55%)	6 (7.5%)	1 (1.31%)	3 (7.14%)	1 (2.94%)		2 (14.28%)	—		
3	11 (9.01%)	10 (12.5%)	2 (2.63%)	8 (19.04%)	—		—	—		

*Local recurrence*										
No	121 (99.19%)	80 (100%)	—	39 (92.86%)	—	—	13	—		—
Yes	1 (0.81%)	—		3 (7.14%)			1			

*Metastasis*										
No	119 (97.55%)	76 (95%)	—	40 (95.24%)	33	22		8 (100%)		—
Yes	3 (2.45%)	4 (5%)		2 (4.76%)	1	1		—		

*Metastasis localization*										
Brain			—	—	—	—	—	—		—
Vertebra	1 (0.81%)	—	—	—	—	—	—	—		—
Supraclavicular	1 (0.81%)	2 (2.5%)	—	—	—	—	—	—		—
Lymph nodes	1 (0.81)	—				—	—			—
Lungs		1 (1.25%)	—	2 (4.76%)	1 (2.94%)	—	—	—		—
Liver		1 (1.25%)	—	—	—	1 (4.34%)	1 (7.1%)	—		—

Abbreviation: MRM, modified radical mastectomy.

**Table 2:** Patients' surgery-specific conditions.

Features	Mucinous (*n* = 122)	Micropapillary (*n* = 80)	Papillary group carcinomas (*n* = 76)	Metaplastic (*n* = 42)	Neuroendocrine (*n* = 34)	Tubular (*n* = 23)	Cribriform (*n* = 14)	Apocrine (*n* = 8)	Acinic cell (*n* = 1)	Secretory (*n* = 1)
*Surgery type*										
Breast-conserving surgery	55 (45.1%)	38 (47.5%)	38 (50%)	11 (26.2%)	13 (38%)	15 (65.2%)	7 (50%)	3 (37.5%)	—	—
Mastectomy	40 (32.8%)	24 (30%)	37 (48.7%)	16 (38.0%)	14 (41%)	3 (13%)	6 (42.9%)	2 (25%)	—	1 (100%)
Mrm	27 (22.1%)	13 (16.5%)	1 (1.3%)	13 (31%)	6 (17%)	2 (8.7%)	1 (7.1%)	2 (25%)	1 (100%)	—
Mastectomy + expander	—	4 (5%)	—	—	1 (4%)	2 (8.7%)	—	—	—	—
Mrm + expander	—	1 (1.5%)	—	—	—	—	—	1 (12.5%)	—	—
Mastectomy + implant				2 (4.8%)	—	1 (4.3%)	—	—	—	—

*Axillary dissection*										
N/A	77 (63.1%)	30 (37.5%)	66 (86.8%)	23 (54.8%)	21 (61.8%)	19 (82.6%)	11 (78.6%)	4 (50%)	—	—
Yes	45 (36.9%)	50 (62.5%)	10 (13.2%)	19 (45.2%)	13 (38.2%)	4 (17.4%)	3 (21.4%)	4 (50%)	1 (100%)	1 (100%)

*Surgical margin status*										
Negative	113 (92.6%)	76 (95%)	69 (90.8%)	39 (92.9%)	32 (94.1%)	21 (91.3%)	14 (100%)	8 (100%)	1 (100%)	1 (100%)
Positive	9 (7.4%)	4 (5%)	7 (9.2%)	3 (7.1%)	2 (5.9%)	2 (8.7%)	—	—	—	—

*Surgical margin positivity*										
Invasive	3 (2.45%)	3 (3.75%)	1 (1.31%)	2 (4.76%)	1 (2.94%)	1 (4.34%)	—	—		—
DCIS	6 (4.9%)	1 (1.25%)	6 (7.89%)	1 (2.38%)	1 (2.94%)	1 (4.34%)	—	—		

*Sentinel lymph node status*										
Negative	73 (59.8%)	29 (36.3%)	63 (82.9%)	24 (57.1%)	19 (55.9%)	19 (82.6%)	11 (78.6%)	4 (50%)	—	—
Positive	19 (15.6%)	33 (41.3%)	9 (11.8%)	3 (7.1%)	8 (23.5%)	2 (8.7%)	2 (14.3%)	2 (25%)	—	1 (100%)
N/A (direct axillary dissection)	30 (24.6%)	18 (22.5%)	4 (5.3%)	15 (35.7%)	7 (20.6%)	2 (8.7%)	1 (7.1%)	2 (25%)	1 (100%)	—
Sentinel lymph node count (mean)	2.87 ± 1.7	2.23 ± 1.28	2.72 ± 1.81	2.85 ± 1.89	2.74 ± 1.87	2.9 ± 1.46	2.69 ± 1.65	2.67 ± 1.36	—	1

Number of positive sentinel lymph nodes (mean)	0.26 ± 0.89	1.05 ± 1.25	1.22 ± 2.22	0.12 ± 0.34	0.44 ± 0.8	0.19 ± 0.67	0.15 ± 0.55	0.33 ± 0.51	—	1

*Extracapsular invasion in axilla-positive patients*										
No	101 (82.8%)	54 (67.5%)	75 98.7%)	37 (88.1%)	30 (88.2%)	23 (100%)	12 (85.7%)	6 (75%)	1 (100%)	—
Yes	21 (17.2%)	26 (32.5%)	1 (1.3%)	5 (11.9%)	4 (11.8%)	0	2 (14.3%)	2 (25%)	—	1 (100%)

Number of axillary dissection lymph nodes in axillary dissection patients (mean)	15.47 ± 7.42	16.72 ± 7.49	20 ± 5.91	18.47 ± 6.01	16.53 ± 5.54	14 ± 1.4	16 ± 2.64	18.5 ± 6.6	27	27

Number of metastatic lymph nodes in axilla dissection (mean)	4.19 ± 4.92	4.5 ± 6.2	3.9 ± 7.09	1.8 ± 2.96	2.92 ± 3.86	1.5 ± 2.1	6.66 ± 4.04	4.50 ± 5	1	0

*Re-excision and complementary mastectomy in breast-conserving surgery*										
No	113 (92.62%)	75 (93.75%)	69 (90.79%)	40 (95.2%)	31 (91.18%)	22 (95.7%)	14 (100%)	8 (100%)	1 (100%)	1 (100%)
Re-excision	5 (4.09%)	1 (1.25%)	4 (5.26%)	1 (2.4%)	2 (5.88%)	1 (4.3%)	—	—	—	—
Complementary mastectomy	2 (1.63%)	3 (3.75%)	1 (1.32%)	—	1 (2.94%)	—	—	—	—	—
Mastectomy after re-excision	1 (0.81%)	1 (1.25%)		1 (2.4%)	—	—	—		—	—
The patient refused re-excision or mastectomy	1 (0.81%)	—	2 (2.63%)	—	—	—	—	—	—	—

Abbreviation: MRM, modified radical mastectomy.

**Table 3:** OS and DFS.

Histopathology	Mean OS (95% CI)	SE	Mean DFS (95% CI)	SE
Mucinous (*n* = 122)	65.774 (62.015–69.533)	1.918	63.880 (59.647–68.112)	2.159
Micropapillary (*n* = 80)	62.496 (60.461–64.532)	1.038	60.113 (56.862–63.365)	1.659
Papillary group carcinomas (*n* = 76)	58.265 (54.647–61.878)	1.845	58.279 (54.676–61.881)	1.838
Metaplastic (*n* = 42)	52.222 (44.428–60.016)	3.976	50.428 (42.309–58.546)	4.142
Neuroendocrine (*n* = 34)	55.860 (49.339–62.381)	3.327	55.853 (49.321–62.385)	3.332
Cribriform (*n* = 14)	58.300 (49.561–67.039)	4.459	55.600 (46.603–64.597)	4.590
Apocrine (*n* = 8)	39.167 (28.730–49.604)	5.325	39.167 (28.730–49.604)	5.325
Tubular (*n* = 23)	⁣^∗^	⁣^∗^	⁣^∗^	⁣^∗^
Acinic cell (*n* = 1)	⁣^∗∗^	⁣^∗∗^	⁣^∗∗^	⁣^∗∗^
Secretory (*n* = 1)	⁣^∗∗∗^			

⁣^∗^Not calculated since all patients survived.

⁣^∗∗^52 months of disease-free follow-up.

⁣^∗∗∗^9 months of disease-free follow-up.

**Table 4:** Patients' clinical and pathological features (papillary group carcinomas and metaplastic carcinoma subgoups).

Clinicopathological variables	Papillary group carcinomas (*n* = 76)			Metaplastic carcinoma (*n* = 42)					
	Invasive solid papillary carcinoma (*n* = 60)	Encapsulated papillary carcinoma with invasion (*n* = 15)	Intraductal papillary adenocarcinoma (*n* = 1)	Metaplastic carcinoma with mesenchymal differentiation (*n* = 21)	Squamous cell carcinoma (*n* = 14)	Spindle cell carcinoma (*n* = 3)	Fibromatoid metaplastic carcinoma (*n* = 2)	Low-grade adenosquamous carcinoma (*n* = 1)	Mixed metaplastic carcinoma (*n* = 1)
*Age*									
Mean	65.67 ± 14.05	64.8 ± 11.4	50	57.19 ± 13.66	59.21 ± 12	55 ± 21.65	36.5 ± 3.5	40	61
Median	61 (28–89)	40 (46–86)		49 (35–84)	32 (44–76)	38 (42–80)	34–39	40	

Radiological tumor size (mean-mm-sd)	25.96 ± 15.97	24.4 ± 11.7	15	34.47 ± 16.79	39.71 ± 17.24	45 ± 31.22	33 ± 9.89	55	37

Pathological tumor size (mean-mm-sd)	28.4 ± 22.56	27.10 ± 14.62	6	34.38 ± 19	40.28 ± 18.20	40 ± 15	40 ± 21	30	35

*T stage*									
Tis	—	—	—	—	—	—	—	—	—
T1	31 (51.7%)	5 (33.3%)	1 (100%)	4 (19%)	1 (7.1%)	—	—	—	—
T2	21 (35%)	8 (53.3%)	—	11 (52.4%)	7 (50%)	2 (66.7%)	1 (50%)	1 (100%)	1 (100%)
T3	8 (13.3%)	2 (13.3%)	—	6 (28.6%)	5 (35.8%)	1 (33.3%)	1 (50%)	—	—
T4	—	—	—	—	1 (7.1%)	—	—	—	—

*Pure or mix pathology*									
Pure	52 (88.1%)	14 (93.3%)	1 (100%)	19 (90.5%)	14 (100%)	3 (100%)	2 (100%)	—	1 (100%)
Mix	7 (11.9%)	1 (6.7%)	—	2 (9.5%)	—	—	—	1 (100%)	—

*Grade*									
Grade 1	5 (8.35)	4 (26.7%)	—	1 (4.8%)	—	—	—	—	—
Grade 2	45 (75%)	10 (66.6%)	1 (100%)	—	—	1 (33.3%)	—	—	—
Grade 3	10 (16.75)	1 (6.7%)	—	20 (95.2%)	14 (100%)	2 (66.7%)	2 (100%)	1 (100%)	1 (100%)

*Hormone receptor*									
Negative	1 (1.7%)	—	—	21 (100%)	14 (100%)	3 (100%)	2 (100%)	—	1 (100%)
Positive	59 (98.3%)	15 (100%)	1 (100%)	—	—	—	—	1 (100%)	—

*Estrogen receptor*									
Negative	—	—	—	21 (100%)	14 (100%)	3 (100%)	2 (100%)	—	1 (100%)
Positive	60 (100%)	15 (100%)	1 (100%)	—	—	—	—	1 (100%)	—

*Progesterone receptor*									
Negative	4 (6.7%)	—	—	21 (100%)	14 (100%)	3 (100%)	2 (100%)	—	1 (100%)
Positive	56 (93.3%)	15 (100%)	1 (100%)	—	—	—	—	1 (100%)	—

Ki-67 (mean-sd)	24.42 ± 17.4	27.82 ± 25.8	—	70.48 ± 19.35	69.64 ± 17.48	60 ± 34.64	92.5 ± 3.5	70	90

*Ki-67*									
≤ 15	24 (40%)	8 (53.3%)	—	—	—	—	—	—	—
> 15	36 (60%)	7 (46.7%)	—	21 (100%)	14 (100%)	3 (100%)	2 (100%)	1 (100%)	1 (100%)
Missing	—	—	1 (100%)	—	—	—	—	—	—

*HER2/neu status*									
Negative	57 (95%)	15 (100%)	1 (100%)	18 (85.7%)	11 (78.8%)	3 (100%)	2 (100%)	1 (100%)	1 (100%)
Positive	3 (5%)	—	—	3 (14.3%)	3 (21.45)	—	—	—	—

*Molecular subtypes*									
Luminal A	57 (95%)	15 (100%)	1 (100%)	—	—	—	—	1 (100%)	—
Luminal B	3 (5%)	—	—	—	1 (7.1%)	—	—	—	—
HER2 positive	—	—	—	2 (9.5%)	2 (14.3%)	—	—	—	—
Triple negative	—	—	—	19 (90.5%)	11 (78.6%)	3 (100%)	2 (100%)	—	1 (100%)

*LCIS*									
No	58 (96.7%)	13 (86.7%)	1 (100%)	1 (4.8%)	13 (92.9%)	2 (66.7%)	2 (100%)	1 (100%)	1 (100%)
Yes	2 (3.3%)	2 (13.3%)	—	20 (95.2%)	1 (7.1%)	1 (33.3%)	—	—	—

*DCIS*									
No	41 (68.3%)	10 (66.7%)	—	16 (76.2%)	7 (50%)	2 (66.7%)	1 (50%)	—	1 (100%)
Yes	19 (31.7%)	5 (33.3%)	1 (100%)	5 (23.8%)	7 (50%)	1 (33.3%)	1 (50%)	1 (100%)	—

*Perineural invasion*									
No	57 (95%)	15 (100%)	1 (100%)	20 (95.2%)	12 (85.7%)	3 (100%)	2 (100%)	1 (100%)	1 (100%)
Yes	3 (5%)	—	—	1 (4.85)	2 (14.3%)	—	—	—	—

*Lymphovascular invasion*									
No	55 (91.7%)	15 (100%)	1 (100%)	17 (81%)	10 (71.4%)	2 (66.7%)	—	—	1 (100%)
Yes	5 (8.3%)	—	—	4 (19%)	4 (28.5%)	1 (33.3%)	2 (100%)	1 (100%)	—

*Synchronicity*									
No malignancy in the contralateral breast	59 (98.3%)	14 (93.3%)	1 (100%)	21 (100%)	13 (92.9%)	3 (100%)	2 (100%)	1 (100%)	1 (100%)
Malignancy in the contralateral breast	1 (1.7%)	1 (6.7%)	—	—	1 (7.1%)	—	—	—	—

*Metachronous*									
No malignancy in the contralateral breast	60 (100%)	15 (100%)	1 (100%)	21 (100%)	14 (100%)	3 (100%)	2 (100%)	1 (100%)	1 (100%)
There is malignancy in the contralateral breast	—	—	—	—	—	—	—	—	—

*Disease focality*									
Unifocal	52 (86.7%)	14 (93.3%)	1 (100%)	21 (100%)	14 (100%)	3 (100%)	2 (100%)	1 (100%)	1 (100%)
Multifocal	8 (13.3%)	1 (6.7%)	—	—	—	—	—	—	—

*Multifocal pathology*									
Same	—	—	—	—	—	—	—	—	—
NST	6 (10%)	1 (7.14%)	—	—	—	—	—	—	—
Other	2 (3.3%)	—	—	—	—	—	—	—	—

*Neoadjuvant status*									
No	57 (95%)	15 (100%)	1 (100%)	15 (71.4%)	11 (78.6%)	3 (100%)	2 (100%)	—	—
Yes	3 (5%)	—	—	6 (28.6%)	3 (21.4%)	—	—	1 (100%)	1 (100%)

*Residual cancer burden system*									
1	—	—	—	1 (4.8%)	—	—	—	—	—
2	1 (1.7%)	—	—	1 (4.8%)	2 (14.3%)	—	—	—	—
3	2 (3.3%)	—	—	4 (19%)	1 (7.1%)	—	—	1 (100%)	1 (100%)

*Local recurrence*									
No	60 (100%)	15 (100%)	1 (100%)	20 (95.2%)	12 (85.7%)	3 (100%)	2 (100%)	1 (100%)	1 (100%)
Yes	—	—	—	1 (4.8%)	2 (14.3%)	—	—	—	—

*Metastasis*									
No	60 (100%)	15 (100%)	1 (100%)	20 (95.2%)	13 (92.9%)	3 (100%)	2 (100%)	1 (100%)	1 (100%)
Yes	—	—	—	1 (4.8%)	1 (7.1%)	—	—	—	—

*Metastasis localization*									
Brain	—	—	—	—	—	—	—	—	—
Vertebra	—	—	—	—	—	—	—	—	—
Supraclavicular	—	—	—	—	—	—	—	—	—
Lymph nodes	—	—	—	—	—	—	—	—	—
Lungs	—	—	—	1 (4.8%)	1 (7.1%)	—	—	—	—
Liver	—	—	—	—	—	—	—	—	—

**Table 5:** Patients' surgery-specific conditions (papillary group carcinomas and metaplastic carcinoma subgroups) (continuation of the table).

Features	Papillary group carcinomas (*n* = 76)			Metaplastic carcinoma (*n* = 42)					
	Invasive solid papillary carcinoma (*n* = 60)	Encapsulated papillary carcinoma with invasion (*n* = 15)	Intraductal papillary adenocarcinoma (*n* = 1)	Metaplastic carcinoma with mesenchymal differentiation (*n* = 21)	Squamous cell carcinoma (*n* = 14)	Spindle cell carcinoma (*n* = 3)	Fibromatoid metaplastic carcinoma (*n* = 2)	Low-grade adenosquamous carcinoma (*n* = 1)	Mixed metaplastic carcinoma (*n* = 1)
*Surgery type*									
Breast-conserving surgery	25 (41.7%)	12 (80%)	1 (100%)	5 (23.8%)	4 (28.6%)	1 (33.3%)	1 (50%)	—	—
Mastectomy	34 (56.7%)	3 (20%)	—	10 (47.6%)	4 (28.6%)	1 (33.3%)	1 (50%)	—	1 (100%)
Mrm	1 (1.6%)	—	—	5 (23.8%)	6 (42.8%)	1 (33.3%)	—	1 (100%)	—
Mastectomy + expander	—	—	—	—	—	—	—	—	—
Mrm + expander	—	—	—	—	—	—	—	—	—
Mastectomy + implant	—	—	—	1 (4.8%)	—	—	—	—	—

*Axillary dissection*									
N/A	52 (86.7%)	13 (86.7%)	1 (100%)	14 (66.7%)	5 (35.7%)	1 (33.3%)	2 (100%)	—	1 (100%)
Yes	8 (13.3%)	2 (13.3%)	—	7 (33.3%)	9 (64.3%)	2 (66.7%)	—	1 (100%)	—

*Surgical margin status*									
Negative	57 (95%)	12 (80%)	—	21 (100%)	12 (85.7%)	3 (100%)	1 (50%)	1 (100%)	1 (100%)
Positive	3 (5%)	3 (20%)	1 (100%)	—	2 (14.3%)	—	1 (50%)	—	—

*Surgical margin positivity*									
Invasive	1 (1.7%)	—	—	—	2 (14.3%)	—	—	—	—
DCIS	2 (3.3%)	3 (20%)	1 (100%)	—	—	—	1 (50%)	—	—

*Sentinel lymph node status*									
Negative	49 (81.7%)	13 (86.7%)	1 (100%)	15 (71.4%)	5 (35.8%)	1 (33.3%)	2 (100%)	—	1 (100%)
Positive	7 (11.6%)	2 (13.3%)	—	1 (4.8%)	1 (7.1%)	1 (33.3%)	—	—	—
N/A (direct axillary dissection)	4 (6.7%)	—	—	5 (23.8%)	8 (57.1%)	1 (33.3%)	—	1 (100%)	—

Sentinel lymph node count (mean)	2.89 ± 1.94	2.1 ± 1.2	2	2.47 ± 1.5	3.5 ± 2	1.5 ± 0.7	2.5 ± 0.7	—	8

Number of positive sentinel lymph nodes (mean)	1.25 ± 2.37	1	—	2	0.25 ± 0.50	0.5 ± 0.7	—	—	—

*Extracapsular invasion in axilla-positive patients*									
No	59 (98.3%)	15 (100%)	1 (100%)	21 (100%)	10 (71.4%)	2 (66.7%)	2 (100%)	1 (100%)	1 (100%)
Yes	1 (1.7%)	—	—	—	4 (28.6%)	1 (33.3%)	—	—	—

Number of axillary dissection lymph nodes in axillary dissection patients (mean)	18.55 ± 5.55	26.5 ± 2.12	—	16.42 ± 5.25	20.77 ± 6.61	19 ± 1.41	—	11	—

Number of metastatic laps in axilla dissection (mean)	1.88 ± 2.71	13 ± 15.5	—	1.85 ± 2.91	2.44 ± 3.57	—	—	1 (100%)	—

*Re-excision and complementary mastectomy in breast-conserving surgery*									
No	57 (95%)	12 (80%)	—	21 (100%)	12 (85.8%)	3 (100%)	1 (50%)	1 (100%)	1 (100%)
Re-excision	2 (3.3%)	2 (13.3%)	—	—	—	—	1 (50%)	—	—
Complementary mastectomy	—	—	1 (100%)	—	1 (7.1%)	—	—	—	—
Mastectomy after re-excision	1 (1.7%)	1 (6.7%)	—	—	1 (7.1%)	—	—	—	—
The patient refused re-excision or mastectomy	—	—	—	—	—	—	—	—	—

Abbreviation: MRM, modified radical mastectomy.

## Data Availability

The data that support the findings of this study are available from the corresponding author upon reasonable request at the following url: https://authorservices.wiley.com/author-resources/Journal-Authors/open-access/data-sharing-citation/data-sharing-policy.html#standardtemplates.
